# Reported self-management of hypertension among adult hypertensive patients in a developing country: a cross-sectional study in a Nigerian tertiary hospital

**DOI:** 10.4314/ahs.v21i3.28

**Published:** 2021-09

**Authors:** Ogban E Omoronyia, Idowu Okesiji, Chiamaka H Uwalaka, Enagu A Mpama

**Affiliations:** 1 Department of Community Medicine, University of Calabar, Calabar, Nigeria; 2 Department of Nursing Science, Lagos State University Teaching Hospital, Ikeja, Nigeria; 3 Nigerian Institute of Medical Research, Lagos, Nigeria

**Keywords:** Hypertension, self-management, self-integration, self-regulation, adherence, Nigeria

## Abstract

**Background:**

Sustained control of blood pressure, is dependent on degree of self-management, which includes self-integration, self-regulation, self-monitoring and adherence to regimen. We assessed the pattern of self-management of hypertension among adult hypertensive patients in a developing country.

**Methods:**

Cross-sectional study design and convenience sampling, was used to recruit adult hypertensive patients, attending Lagos State University Teaching Hospital, Lagos, Nigeria. Interviewer-administered questionnaire was used to obtain data on self-management components. SPSS version 21.0 was used to analyze data, with p-value set at 0.05.

**Result:**

One hundred and seven (107) respondents, had mean age of 49.0 ± 12.0 years. Mean value for self-management was 3.15 ± 0.55, comprising self-integration (3.06 ± 0.36), self-regulation (3.32 ± 0.63), self-monitoring (3.29 ± 0.84) and adherence to regimen (3.15 ± 0.55). Most components of self-management, had high levels of mean score. Respondents that were less than 40 years, compared with those that were more than 40 years, had greater mean values for self-integration (3.37 vs 3.05), but significantly lesser values for all other components (p < 0.05).

**Conclusion:**

Young hypertensives had poor levels of most components of self-management. There is urgent need for health educational programs on self-management of hypertension among young people in sub-Saharan Africa.

## Introduction

Hypertension is a silent killer, and leading cause of morbidity and mortality globally^1^. At least one in every four adults, is hypertensive in the sub-Saharan African region, which has one of the weakest health systems^2^. Sedentary lifestyle, poor dietary habits, psychosocial stressors and other modifiable risk factors for its development and progression, are also on the rapid rise and therefore contribute to high burden of disease, especially arising from cardiovascular complications^2^. In developing countries with mostly out-of-pocket payment for health services, several households have become poor or even extremely poor due to morbidity and mortality related to hypertension^3^.

Unfortunately, there is yet no cure for essential hypertension, which is the most common form of the chronic disease^4^. Best practice for its prevention and control, remains regular screening for early diagnosis, followed by non-pharmacologic and pharmacologic treatment^5^. This practice requires involvement of hypertensive patients, in the initiation and sustenance of measures aimed at lifelong control of their blood pressure, and prevention of cardiovascular complications^5^,^6^. In particular, uncontrolled hypertension has been associated with patients' non-adherence to recommended self-management practices^7^.

Key aspect of patient involvement in self-management of their disease, is the integration of dietary, physical exercise, stress management and other lifestyle changes into their daily living^8^. Such self-integration contributes significantly to blood pressure control, and minimizes risk of occurrence and progression of complications. Other aspects of patient involvement include, active effort at monitoring and attainment of set blood pressure goals (self-regulation), consistency in follow-up appointment visits (self-monitoring), adherence to prescribed regimen, and effective interaction with caregiving professionals and significant others. These components of self-management are key determinants of successful lifelong control of blood pressure^8^. They should therefore be assessed regularly, towards provision of effective individualized care for hypertensive patients^5^.

There are objective means of assessing these components of self-management of hypertension using structured questionnaire8. Mean scores and levels can be obtained for each component, and compared across timeframes for effective patient management, or between study populations in diverse global settings8. In a survey among hypertensive patients in Bangladesh, moderate levels of each component of self-management was found, with overall mean value of 2.55 ± 0.479. These levels reflect behaviours related to self-management, and may be useful for monitoring and follow-up care of patients. A facility-based cross-sectional study of self-management behaviors among hypertensive patients in Egypt, found 56.7% prevalence of negative behaviors towards hypertension^10^. Most of the respondents in the study were however illiterate housewives, which may not be typically representative of hypertensive population in developing country setting. Similar study in Ethiopia reported 59.4% prevalence of proper self-care management of hypertension^11^.

Unfortunately, there is paucity of research on self-management of hypertension in Nigeria, which has one of the highest burden of cardiovascular diseases in sub-Saharan Africa^8^. Independent studies among hypertensive patients, in South West^12^ and North West^13^ Nigeria, focused on social support and adherence to medications, without assessing each of the components of self-management of hypertension. Better understanding of the pattern of each component of self-management is key, for improvement in best practice of engaging patients in control of their blood pressure^6^, ^14^. This study was aimed at assessing pattern of self-management, among hypertensive patients in a developing country setting.

## Methods

Cross-sectional study design was utilized, and respondents were recruited by convenience sampling from Lagos State University Teaching Hospital (LASUTH), Lagos, Nigeria. Sample comprised attendees of medical out-patient clinic LASUTH, who had been diagnosed of hypertension. Sample size of 106 was calculated using Yamane formula^15^ n = N / 1+ N (e)^2^, where N is estimated monthly clinic uptake of 125 hypertensive patients, e is error margin of 0.05 and assuming a non-response rate of 10%. Consenting adult hypertensive patients that were at least 20 years old were eligible to participate. Patients that had been diagnosed of hypertension within 12 months were excluded. Also, patients with confirmed diagnosis, as well as those that were being investigated for secondary hypertension were excluded from the study. Patients who were healthcare professionals were excluded from participating.

Pretested, interviewer-administered, structured questionnaire, was used to obtain quantitative data, on respondents' socio-demographic variables, the patterns and factors associated with self-management of hypertension. Questionnaire used in this study, was an adaptation of the validated tool used for assessment of self-management of diabetes^15^, and previously used in similar developing country setting^9^. It was pretested before use for data collection. The 35-item questionnaire, had five components comprising 10, 7, 9, 4 and 5 questions for self-integration, self-regulation, interpersonal relationship with professionals, self-monitoring and adherence to regimen, respectively. Each question had a 4-point Likert scale of responses, consisting of ‘never’, ‘rarely’, ‘sometimes’, and ‘always’.

SPSS was used to enter and analyze data. Frequency distribution of responses to each of the questions was presented in tabular form. Score of 1, 2, 3 and 4, were allocated to ‘never’, ‘rarely’, ‘sometimes’, and ‘always’ Likert scales, respectively. Questions that imply inappropriate practice such as questions 5 and 10 were reverse-scored. Responses to each item was scored, with mean scores obtained for each component, and overall mean score for self-management. Mean scores were categorized into low, moderate, and high levels of self-managementcorresponding to 1.00–2.00, 2.01–3.00, and 3.01–4.00, respectively. Independent t-test and Analysis of Variance (ANOVA), were employed as inferential statistics, to compare mean scores between categorical variables. P-value was set at 0.05. Ethical approval was obtained from the research ethics committee, of Lagos State University Teaching Hospital, Nigeria. Informed and written consent was obtained from respondents before data collection.

## Results

One hundred and seven (107) respondents gave complete data, with mean age of 49.0 ± 12.0 years ranging from 20 to 69 years. Female: male ratio was 1.2:1, and most respondents (77.6%) were 40 years or older (table 1). Eighty-eight respondents (82.3%) had at least secondary level of education, while 53.3% were either civil or public servants. Most respondents were married (57.0%), never smoked (92.5%) nor consumed alcoholic beverages (86.0%) (table 1).

**Table 1:** Socio-demographic characteristics (N=107)

Variable	Frequency	Percentage
**Gender**		
Male	48	44.9
Female	59	55.1
**Age groups (in years)**		
20–29	6	5.6
30–39	18	16.8
40–49	41	38.3
50–59	20	18.7
>60	22	20.6
**Level of education**		
None	6	5.6
Primary	13	12.1
Secondary	38	35.6
Tertiary	50	46.7
**Marital status**		
Married	61	57.0
Single	16	15.0
Divorced	14	13.1
Separated	10	9.3
Widowed	6	5.6
**Occupation**		
Civil / Public servant	57	53.3
Business/trader	33	30.8
Student	12	11.2
Unemployed	5	4.7
**Smoking status**		
Never	99	92.5
previously smoked but quit	4	3.7
Still smoking	4	3.7
**Drinking status**		
Never	92	86.0
previously consumed alcohol but quit	11	10.3
Still consuming alcoholic beverages	4	3.7

Table 2 shows distribution of responses to questions regarding self-integration and self-regulation aspects of self-management of hypertension. Self-integration questions with high frequency of ‘always’ response were consumption of fruits, vegetables and legumes (84.1%), stress management through music, rest, and relationships (82.2%), and consideration of food portion size (76.6%). Most respondents either sometimes or always decreased consumption of fatty diets (87.9%), considered their blood pressure in making food choices (85.1%), exercised regularly (88.8%), and fitted their daily routine considering their hypertension status (79.5%). However, (89.1%) never (62.6%) or rarely (6.5%) thought hypertension to be part of their lives. Also, approximately half of respondents (52.3%) never (3.7%) or rarely (48.6%) choose less salty food, while over a third (36.5%) sometimes (26.2%) or always (10.3%) used extra salt for seasoning (table 2). Considering self-regulation, most respondents could sometimes or always recognize symptoms of high (89.7%) and low (78.5%) blood pressures (as described for them during data collection), manage their symptoms properly (87.8%), and set goals for blood pressure control (87.0%) (table 2).

**Table 2:** Frequency distribution of responses to components of self-integration and regulation (N=107)

SN	Variable	Never	Rarely	Sometimes	Always
		n (%)	n (%)	n (%)	n (%)
**Self-Integration**					
1	Do you consider about food portions and choices whenever you have to eat food?	2 (1.9)	4 (3.7)	19 (17.8)	82 (76.6)
2	Have you eaten fruits, vegetable, grains, and beans more than when you did not have hypertension?	4 (3.7)	4 (3.7)	9 (8.4)	90 (84.1)
3	Have you decreased food that contains high saturated fat (e.g., coconut oil, palm oil, cheese etc.) since being diagnosed?	7 (6.5)	6 (5.6)	69 (64.5)	25 (23.4)
4	Do you think of your blood pressure when making food choices?	4 (3.7)	12 (11.2)	66 (61.7)	25 (23.4)
5	Do you choose less salty food?	4 (3.7)	52 (48.6)	13 (12.1)	38 (35.5)
6	Do you exercise to reduce your weight (such as walking, jogging (running), and/ or cycling for about 30–60 minutes/session at least thrice weekly)?	7 (6.5)	5 (4.7)	26 (24.3)	69 (64.5)
7	Do you think that your hypertension is a part of your life?	67 (62.6)	7 (6.5)	17 (15.9)	16 (15.0)
8	Have you made your routine to fit with things you have to do for your hypertension (such as my work and my doctor visit)?	9 (8.4)	13 (12.1)	11 (10.3)	74 (69.2)
9	Have you tried to control your stress by listening to music, taking rest, talking with your family or friends, etc.?	5 (4.7)	3 (2.8)	11 (10.3)	88 (82.2)
10	Do you use extra salt to season food since being diagnosed?	63 (58.9)	5 (4.7)	28 (26.2)	11 (10.3)
**Self-regulation**					
11	Have you recognized signs and symptoms of high blood pressure (e.g. headache, chest pain, blurry vision / visual loss)?	10 (9.3)	1 (0.9)	14 (13.1)	82 (76.6)
12	Have you managed your hypertensive signs and symptoms properly?	8 (7.5)	5 (4.7)	18 (16.8)	76 (71.0)
13	Have you recognized signs and symptoms of low blood pressure (e.g. dizziness, light headedness, fatigue, blurry vision)?	17 (15.9)	6 (5.6)	18 (16.8)	66 (61.7)
14	Have you made your goal towards your blood pressure control	7 (6.5)	7 (6.5)	19 (17.8)	74 (69.2)
15	Have you made your action plan to achieve your goal towards your blood pressure control?	8 (7.5)	8 (7.5)	23 (21.5)	68 (63.6)
16	Have you compared your current blood pressure level with the target (desired, controlled) level?	6 (5.6)	4 (3.7)	76 (71.0)	21 (19.6)
17	Have you managed situations that may increase your blood pressure?	8 (7.5)	3 (2.8)	77 (72.0)	19 (17.8)

Table 3 shows distribution of responses to questions regarding interaction with professionals, self-monitoring and adherence to treatment regimen. Area with commonest response of ‘never’ for integration with professionals, was helping health workers find out reasons for poor control of blood pressure (69.2%). Most respondents, either sometimes or always discussed flexibility of treatment plans with doctor (81.4%), suggested change in treatment plan (71.1%), asked for clarity from doctors or nurses (81.3%), asked for where they could learn more about hypertension (73.8%), and how their friends managed their blood pressure (73.8%). Concerning self-monitoring, most respondents (72.0%) always checked their blood pressure with their doctor when they felt sick. However, 17.7% and 28.9% never or rarely checked their blood pressure when they experiences symptoms of high and low blood pressures, respectively. Approximately one-fifth of respondents (21.5%) did not adhere strictly to their prescribed antihypertensive medications (table 3).

**Table 3:** Frequency distribution of responses to components of interaction, monitoring and adherence (N=107)

SN	Variable	Never	Rarely	Sometimes	Always
		n (%)	n (%)	n (%)	n (%)
**Interaction with professionals and significant others**					
18	Have you discussed the flexibility of treatment plan with your doctor or nurses regarding my treatment plan?	13 (12.1)	7 (6.5)	16 (15.0)	71 (66.4)
19	Have you suggested to your doctor to change the treatment plan if you may not be able to conform with the plan?	27 (25.2)	4 (3.7)	8 (7.5)	68 (63.6)
20	Have you asked your doctor or nurses when there are things you do not understand?	12 (11.2)	8 (7.5)	17 (15.9)	70 (65.4)
21	Have you helped your doctor or nurses to find why your blood pressure is not well controlled?	74 (69.2)	7 (6.5)	11 (10.3)	15 (14.0)
22	Have you discussed with your doctor or nurses when your blood pressure is too high or too low?	20 (18.7)	6 (5.6)	15 (14.0)	66 (61.7)
23	Have you asked your doctor or nurses where you can learn more about my hypertension?	26 (24.3)	2 (1.9)	17 (15.9)	62 (57.9)
24	Have you asked others (such as friends, neighbors, other patients) for their help with your high blood pressure?	21 (19.6)	8 (7.5)	59 (55.1)	19 (17.8)
25	Have you asked others (such as friends, neighbors, other patients) for their help in controlling your blood pressure?	29 (27.1)	7 (6.5)	62 (57.9)	9 (8.4)
26	Have you asked others (such as friends, neighbors, other patients) how they have managed or what techniques they have used to control their high blood pressure?	23 (21.5)	5 (4.7)	23 (21.5)	56 (52.3)
**Self-monitoring**					
27	Have you checked or have visited your doctor to check your blood pressure when you have experienced signs and symptoms suggestive of uncontrolled high blood pressure (e.g. headache, chest pain, blurry vision / visual loss)?	17 (15.9)	3 (2.8)	12 (11.2)	75 (70.1)
28	Have you checked or have visited your doctor to check your blood pressure when you felt sick?	7 (6.5)	6 (5.6)	17 (15.9)	77 (72.0)
29	Have you checked or have visited your doctor to check your blood pressure when you experienced signs and symptoms suggestive of low blood pressure (e.g. dizziness, lightheadedness, fatigue, blurry vision)?	27 (25.2)	4 (3.7)	13 (12.1)	63 (58.9)
30	Have you checked your blood pressure on a regular basis to help make your self-management decision	8 (7.5)	6 (5.6)	49 (45.8)	44 (41.1)
**Adherence to recommended regimen**					
31	Have you strictly taken your antihypertensive medications?	19 (17.8)	4 (3.7)	3 (2.8)	81 (75.7)
32	Have you taken the right amount of your antihypertensive medications	15 (14.0)	5 (4.7)	3 (2.8)	84 (78.5)
33	Have you taken your antihypertensive medications at the right times	15 (14.0)	0 (0.0)	11 (10.3)	81 (75.7)
34	Have you visited your doctor as scheduled?	12 (11.2)	6 (5.6)	7 (6.5)	82 (76.6)
35	Have you followed your doctors' or nurses' advice regarding your blood pressure control?	14 (13.1)	0 (0.0)	0 (0.0)	93 (86.9)

Table 4 shows distribution of mean scores and levels for each of the components of self-management of hypertension. Except for interaction with professionals and others which had moderate level, all other components as well as total self-management had high levels of mean score (table 4). Table 5 shows factors associated with mean levels for of each component of self-management of hypertension. Mean values for all components, as well as overall self-management of hypertension, were not significantly different comparing both gender (p>0.05). Respondents that were less than 40 years, compared with those that were more than 40 years, had greater mean values for self-integration (3.37 vs 3.05), but significantly lesser values for all other components (p < 0.05). Compared with other occupations, students had significantly lesser mean values for self-regulation and self-monitoring (p<0.05). Also, smokers and those who consumed alcoholic beverages had significantly lesser mean values for adherence to antihypertensive regimen (p<0.05). Level of education and marital status were no associated with any of the components of self-management (p>0.05).

**Table 4:** Distribution of level and mean scores for each component of self-management (N=107)

Variable	Low n (%)	Moderate n (%)	High n (%)	Mean (SD)	Mean Level
Self-integration	0 (0.0)	48 (44.9)	59 (55.1)	3.06 (0.36)	High
Self-regulation	5 (4.7)	21 (19.6)	81 (75.7)	3.32 (0.63)	High
Interaction with health professionals and others	20 (18.7)	29 (27.1)	58 (54.2)	2.89 (0.79)	Moderate
Self-monitoring	13 (12.1)	15 (14.0)	79 (73.8)	3.29 (0.84)	High
Adherence to recommended regimens	14 (13.1)	8 (7.5)	85 (79.4)	3.15 (0.55)	High
Total (overall self-management)	6 (5.6)	22 (20.6)	79 (73.8)	3.15 (0.55)	High

**Table 5:** Factors associated with mean levels of each component of self-management (N=107)

Variable	Self-integration Mean (SD)	Self-regulation Mean (SD)	Interaction Mean (SD)	Self-monitoring Mean (SD)	Adherence Mean (SD)	Total (Self-management) Mean (SD)
**Gender**						
Male	2.98 (0.33)	3.30 (0.70)	2.96 (0.72)	3.31 (0.80)	3.59 (0.94)	3.16 (0.55)
Female	3.13 (0.36)	3.34 (0.58)	2.83 (0.85)	3.27 (0.88)	3.39 (1.02)	3.15 (0.55)
t-test p-value	0.072	0.721	0.413	0.811	0.294	0.893
**Age groups** (in yrs.)						
40 or less	3.37 (0.52)	2.55 (1.20)	2.10 (1.10)	2.31 (1.31)	2.25 (1.33)	2.63 (1.10)
>40	3.05 (0.39)	3.32 (0.43)	2.85 (0.71)	3.22 (0.70)	3.55 (0.87)	3.24 (0.48)
t-test p-value	0.001*	0.000*	0.010*	0.011*	0.001*	0.012*
**Level of education**						
Primary or less	3.06 (0.33)	3.45 (0.39)	3.04 (0.65)	3.45 (0.73)	3.74 (0.70)	3.27 (0.36)
Secondary or greater	3.06 (0.37)	3.30 (0.67)	2.85 (0.82)	3.25 (0.87)	3.42 (1.03)	3.13 (0.58)
t-test p-value	0.992	0.341	0.364	0.363	0.212	0.301
**Marital status**						
Married	3.01 (0.36)	3.31 (0.59)	2.83 (0.79)	3.20 (0.80)	3.40 (1.08)	3.10 (0.54)
Unmarried	3.13 (0.53)	3.34 (0.67)	2.96 (0.80)	3.40 (0.90)	3.58 (0.85)	3.22 (0.55)
t-test p-value	0.082	0.831	0.404	0.243	0.372	0.261
**Occupation**						
Civil/public servant	3.01 (0.37)	3.35 (0.56)	2.85 (0.73)	3.31 (0.69)	3.49 (1.06)	3.14 (0.53)
Business/trader	3.10 (0.20)	3.45 (0.50)	3.15 (0.61)	3.48 (0.77)	3.69 (0.68)	3.31 (0.42)
Student	3.08 (0.55)	2.78 (1.05)	2.38 (1.08)	2.60 (1.26)	2.95 (1.10)	2.77 (0.81)
Unemployed	3.38 (0.38)	3.54 (0.23)	2.80 (0.62)	3.40 (0.70)	3.36 (1.32)	3.26 (0.28)
F-test p-value	0.182	0.031*	0.072	0.041*	0.283	0.061
**Smoking status**						
Yes	2.70 (0.59)	2.86 (0.75)	2.89 (0.22)	3.00 (0.89)	2.50 (1.73)	2.79 (0.64)
No	3.07 (0.34)	3.34 (0.62)	2.89 (0.81)	3.30 (0.84)	3.52 (0.94)	3.17 (0.54)
t-test p-value	0.042*	0.131	0.992	0.494	0.042*	0.173
**Drinking status**						
Yes	2.90 (0.42)	2.96 (0.87)	2.72 (1.16)	3.12 (0.88)	2.50 (1.73)	2.83 (0.42)
No	3.07 (0.36)	3.34 (0.62)	2.89 (0.78)	3.29 (0.84)	3.52 (0.94)	3.17 (0.54)
t-test p-value	0.372	0.251	0.672	0.701	0.044*	0.241

## Discussion

This is one of few studies that assessed pattern of self-management of hypertension in Nigeria, which has one of the highest burden of the chronic disease globally. Unlike the few previous studies in Nigeria, this study assessed each component of self-management among wider age-range of hypertensive patients, including young people who now constitute more than two-thirds of the nation's population^17^. Hence, though most respondents were older adults, a significant proportion were 40 years old or younger, and therefore potentially bear much of the dsease burden in the region. This finding is in keeping with estimates of increasing trend of hypertension across all ages, without exception to young people^18^. Considering the chronic life-long nature of hypertension, this younger sub-population are at potentially higher risk of developing complications, especially if self-management remains unsatisfactory^19^. The South West region where the study was located, also has one of the highest prevalence rates and burden of disease due to hypertension in Nigeria18. This is key, considering that the study area and region is one of the most populated in Nigeria^20^, ^21^, with high proportion of young people, who are potentially exposed to diverse psychosocial, occupational, environmental and other unknown risk factors of the chronic disease^22^. Therefore, local and regional health systems in developing countries need to anticipate high need for chronic disease care in the near future when these young adults may be developing complications^19^, ^23^.

Except for few notable areas in each component, self-management of hypertension was generally satisfactory in this study. This finding may be due to years of exposure to passive and active health education and counselling in the study area, where patients have been reported to be quite satisfied with care given towards their blood pressure control^24^. Considering poor doctor-patient ratio, physician burnout and potentially short duration of clinic sessions in Nigeria^25^, ^26^, other sources of health information may have contributed to this high proportion with satisfactory self-management practice^27^. These sources, which include mass media, internet, and discussion with family and friends, may therefore be usefuchannels for cardiovascular health communication in developing countries^28^, ^29^.

Most respondents did not consider hypertension to be part of their lives. In other words, there was high prevalence of non-acceptance of the philosophy of lifelong nature of essential hypertension. This position by respondents, may result from their level of spirituality or belief in possibility of cure for the chronic disease some time sooner or later during their life-course, especially with use of complementary and alternative medicine^30^, ^31^. It may be difficult or require longer duration of counselling, for hypertensive patients to come to terms with the permanent nature of their disease and need for life-long compliance with taking medications daily and possible polypharmacy subsequently^32^. Earlier adjustment to this reality, may be useful towards ensuring long-term or lifelong adherence to medications and avoidance of complications^32^.

This study found poor attitude and practice of salt consumption, among significant proportion of respondents. This practice may potentially reverse gains obtained from beneficial lifestyle modifications. In other words, the high blood pressure effects of excessive salt intake, may neutralize beneficial blood pressure lowering effects of regular exercise, proper dietary habits and stress management. Therefore, excessive salt consumption, may be the singular reason for poor blood pressure control, despite adherence to medications and practice of other lifestyle measures^33^, ^34^. Difficulty in compliance or adherence to low salt consumption, may be due to general belief in its necessity for pleasurable taste of food and overall nutritive value of diets^35^. Unfortunately there may be difficulty in providing practical communication, and quantitative demonstration of limits of daily salt consumption during busy clinic counselling sessions^36^. Adherence to daily limits may also be difficult for hypertensive patients that regularly eat out or consume fast foods^36^.

Self-integration was found to be significantly better among females compared with males. In other words, female hypertensive patients are better at ensuring practice of preventive measures of proper diet, exercise, and stress management, compared with males. This finding suggests that females may be more conscious of the beneficial effects of these measures, as well as severity of non-compliance, in tune with health belief model of health behaviour. Added benefit of potentially better cosmetic looks from weight loss or maintenance and appropriate diet and other lifestyle modifications, may be more appealing as incentive for females compared with males^37^, ^38^. Also, through the years, females may be more sedentary and gain additional weight especially following childbirth. They may therefore be more desirous of self-integration towards restoration to pre-pregnancy body weight and health status^38^.

During collection of data on self-monitoring component, the symptoms suggestive of high and low blood pressure were described to respondents. Most respondents reported recognizing and managing themselves or knowing what to do, when they had these symptoms. However, considering that these symptoms are quite non-specific, the responses may not be considered sufficiently reliable. Yet, while their report of recognition and knowing what to do may appear satisfactory, it may suggest poor control of blood pressure. Well controlled blood pressure, should be devoid of symptoms of high blood pressure. These symptoms may occur due to poor timing of intake of prescribed antihypertensive, as well as potential interaction of the medications with diet, and the influence of other daily activities^39^–^41^. Therefore, on the contrary, respondents that do not recognize symptoms of high blood pressure may be the ones with more effective control. Alternating sequence of presence and resolution of symptoms, may be indicating disease progression that may culminate in subsequent major cardiovascular event such as stroke and heart attack^19^, ^41^.

Additional finding in this study was significantly lower mean values of all components of self-management among respondents that were within 40 years old, compared with those that were older. This suggest poor attitude and practice of preventive measures among younger hypertensive patients. Their potential surprise and stigma due to relatively early diagnosis, belief in its reversibility, and perception of bleak future health status, may all contribute to inconsistencies in initial adoption of preventive measures^42^. Unfortunately, long-term inconsistencies in self-management may contribute to early onset of cardiovascular complications and high disease burden^42^.

This study has notable limitations. Sample size of 107 hypertensive patents may be quite small and therefore not representative of general population of hypertensive adults in the study setting. Considering that data collection was through interviewer administration of questionnaire, there is possibility of social desirability bias in response to smoking and drinking status. Hence, the true proportion of smokers and drinkers may have been higher than reported in this study. Responses to some questions assessing self-regulation and self-monitoring may also have been under-reported or misunderstood, warranting caution in interpretation of findings, especially for items 12, 27 and 29 (tables 2 and 3). For instance, though trained research assistants provided examples of symptoms suggestive of uncontrolled hypertension and hypotension to enable appropriate response, these symptoms are quite non-specific. Besides non-specificity, there may be differential non-response bias in response, comparing respondents who truly had, and those who did not have these symptoms. Yet, these statements are generally acceptable and validated (though subjective) means of assessing for degree of self-monitoring of chronic cardiometabolic diseases^16^, ^33^.

## Conclusion

This study found high level of self-management of hypertension overall, and for each of the components, except for interaction with professionals, which was within moderate level. Despite this generally satisfactory level of self-management, young people within 40 years, had poor levels of all components of self-management. There is also poor attitude and practice ofalt consumption, which may potentially reverse gains in practice of other preventive measures. These findings have implications for prevention and control of the increasing trend in burden of disease due to hypertension even among young people in developing countries. Other sociodemographic characteristics including gender, level of education and occupation were not associated with self-management and its components.

## Recommendations

There is need for regular assessment of level of self-management of hypertension, as part of routine care during clinic visits, with priority focus on young people. Following such assessment, individualized health education and counseling may be more effective, towards cardiovascular disease prevention in developing country settings. Further research in other settings is recommended, especially considering the limitations identified in this study. Hence, a much larger sample size should be studied, preferably in diverse study settings to improve on generalizability of the findings to population of hypertensive patients at least in the study area and region. Considering that instrument used in this study was originally designed for diabetic patients, further studies should include development and validation of tool(s) for assessment of self-management, specifically suitable for hypertensive patients. Further studies should also consider including use of qualitative methods for assessment of non-verbal aspects of attitude and perception towards self-management of hypertension.
